# TRPC4α and TRPC4β Similarly Affect Neonatal Cardiomyocyte Survival during Chronic GPCR Stimulation

**DOI:** 10.1371/journal.pone.0168446

**Published:** 2016-12-19

**Authors:** Nadine Kirschmer, Sandra Bandleon, Viktor von Ehrlich-Treuenstätt, Sonja Hartmann, Alice Schaaf, Anna-Karina Lamprecht, Erick Miranda-Laferte, Tanja Langsenlehner, Oliver Ritter, Petra Eder

**Affiliations:** 1 Department of Internal Medicine I, University Hospital Würzburg, Würzburg, Germany; 2 Comprehensive Heart Failure Center Würzburg, University Hospital Würzburg, Würzburg, Germany; 3 Center for Pharmacometrics and Systems Pharmacology, Department of Pharmaceutics, College of Pharmacy, University of Florida, Orlando, United States of America; 4 Institute of Physiology, University of Würzburg, Würzburg, Germany; 5 Department of Therapeutic Radiology and Oncology, Medical University of Graz, Graz, Austria; 6 Department of Cardiology and Pulmology, Brandenburg Medical School, University Hospital Brandenburg, Brandenburg, Germany; Indiana University School of Medicine, UNITED STATES

## Abstract

The Transient Receptor Potential Channel Subunit 4 (TRPC4) has been considered as a crucial Ca^2+^ component in cardiomyocytes promoting structural and functional remodeling in the course of pathological cardiac hypertrophy. TRPC4 assembles as homo or hetero-tetramer in the plasma membrane, allowing a non-selective Na^+^ and Ca^2+^ influx. Gαq protein-coupled receptor (GPCR) stimulation is known to increase TRPC4 channel activity and a TRPC4-mediated Ca^2+^ influx which has been regarded as ideal Ca^2+^ source for calcineurin and subsequent nuclear factor of activated T-cells (NFAT) activation. Functional properties of TRPC4 are also based on the expression of the TRPC4 splice variants TRPC4α and TRPC4β. Aim of the present study was to analyze cytosolic Ca^2+^ signals, signaling, hypertrophy and vitality of cardiomyocytes in dependence on the expression level of either TRPC4α or TRPC4β. The analysis of Ca^2+^ transients in neonatal rat cardiomyocytes (NRCs) showed that TRPC4α and TRPC4β affected Ca^2+^ cycling in beating cardiomyocytes with both splice variants inducing an elevation of the Ca^2+^ transient amplitude at baseline and TRPC4β increasing the Ca^2+^ peak during angiotensin II (Ang II) stimulation. NRCs infected with TRPC4β (Ad-C4β) also responded with a sustained Ca^2+^ influx when treated with Ang II under non-pacing conditions. Consistent with the Ca^2+^ data, NRCs infected with TRPC4α (Ad-C4α) showed an elevated calcineurin/NFAT activity and a baseline hypertrophic phenotype but did not further develop hypertrophy during chronic Ang II/phenylephrine stimulation. Down-regulation of endogenous TRPC4α reversed these effects, resulting in less hypertrophy of NRCs at baseline but a markedly increased hypertrophic enlargement after chronic agonist stimulation. Ad-C4β NRCs did not exhibit baseline calcineurin/NFAT activity or hypertrophy but responded with an increased calcineurin/NFAT activity after GPCR stimulation. However, this effect was not translated into an increased propensity towards hypertrophy but rather less hypertrophy during GPCR stimulation. Further analyses revealed that, although hypertrophy was preserved in Ad-C4α NRCs and even attenuated in Ad-C4β NRCs, cardiomyocytes had an increased apoptosis rate and thus were less viable after chronic GPCR stimulation. These findings suggest that TRPC4α and TRPC4β differentially affect Ca^2+^ signals, calcineurin/NFAT signaling and hypertrophy but similarly impair cardiomyocyte viability during GPCR stimulation.

## Introduction

Ion channels of the Transient Receptor Potential Channel-canonical type ‘C’ (TRPC) family have been suggested as key players in several cardiac disease conditions [1,2]. They comprise 7 subunits (TRPC1-7) which are all expressed in the heart depending on the developmental stage, species and occurrence of disease [1,3]. They are assembled as tetramers in the sarcolemma of cardiomyocytes, are activated in conditions of Gαq protein-coupled receptor (GPCR) stimulation and allow a non-selective Na^+^/Ca^2+^ influx [1]. These characteristics have been considered as an ideal combination for activating Ca^2+^-dependent signaling pathways and promoting cardiac disease. For example, transgenic mice with a cardiac-specific overexpression of TRPC3 exhibit an enhanced Ca^2+^ influx after concomitant angiotensin II (Ang II) stimulation which causes an exaggerated hypertrophic growth and cardiomyopathy [4]. Similarly, hypertrophic mouse cardiomyocytes isolated after transverse aortic constriction (TAC) surgery exhibit a sustained Ca^2+^ influx which can be blocked by the expression of dominant negative (dn)-TRPC3 and TRPC6 mutants. This inhibition is coupled with less calcineurin and nuclear factor of activated T-cells (NFAT) activation, an attenuated hypertrophic phenotype as well as an improvement of cardiac function [5]. Also, the lack of TRPC1 function in the heart resulted in reduced calcineurin/NFAT signaling, prevented pathological cardiac hypertrophy but preserved contractility [6].

Recently, TRPC4 has caught attention as a crucial component affecting cardiomyocyte pathophysiology. Inhibition of TRPC4 with a truncated dn-TRPC4 mutant resulted in less pathological hypertrophy after TAC treatment [5]. This effect was associated with the absence of a store-operated Ca^2+^ entry (SOCE) in cardiomyocytes from dn-TRPC4 transgenic mice compared to wild-type cardiomyocytes after chronic pressure overload treatment. Another study demonstrated that up-regulation of TRPC4 in the course of myocardial infarction (MI) could cause excessive sarcoplasmic reticulum (SR) Ca^2+^ loading and Ca^2+^ leakage which might contribute to a depressed contractile reserve during disease [2]. Interestingly, inhibition of TRPC4 is also connected with a reduced TRPC3-dependent Ca^2+^ influx in cardiomyocytes [5]. Another recent study suggests a combined activity of TRPC4 with TRPC1 causing a background Ca^2+^ entry, which causes an elevation of diastolic and systolic Ca^2+^ levels and induces reactive signaling and cardiac hypertrophy [7]. These findings might indicate some degree of heteromerization between TRPC4 and other TRPC isoforms in the heart.

The expression of different splice variants might constitute another critical feature for TRPC4–dependent effects on heart disease. Several TRPC4 splice variants have been found, including TRPC4α and TRPC4β which are the most abundantly expressed and best described isoforms [8,9]. They share a high sequence homology but differ within a stretch of 84 amino acids (AA; Δ84AA) in their C termini. This Δ84AA region is only present in TRPC4α and might determine functional differences between TRPC4α and TRPC4β. For example, it has been suggested that the Δ84AA region functions as an autoinhibitory domain limiting the responsiveness of TRPC4α to receptor activation [8]. Moreover, a phosphatidylinositol 4,5-bisphosphate (PIP2) binding region has been found which exclusively inhibits TRPC4α in a highly selective manner [10]. Also in cardiomyocytes, the Δ84AA C terminal region was suggested as critical interaction domain that mediates the complex formation between TRPC4α but not TRPC4β with phospholipase Cβ1b (PLCβ1b) within a signaling complex in the sarcolemma and subsequent spontaneous effects of TRPC4α on cardiomyocyte hypertrophy [11].

In the present study we pursued the hypothesis of TRPC4α and TRPC4β as important determinants affecting TRPC4 channel function in cardiomyocytes. Our findings indicate that the overexpression of human TRPC4α induces a baseline hypertrophic growth of cardiomyocytes which is associated with elevated Ca^2+^ transient peaks and an enhanced calcineurin/NFAT activity. During chronic agonist stimulation, TRPC4α does not further advance hypertrophy but rather induces apoptosis and lowers cell viability. Down-regulation of endogenous TRPC4α induces the opposite phenotype of NRCs by decreasing the cell size at baseline but promoting hypertrophy after agonist stimulation. The overexpression of murine TRPC4β does not induce calcineurin/NFAT activation or hypertrophy at baseline but induces an enhanced calcineurin/NFAT activity after GPCR stimulation. This effect does not increase cardiomyocyte hypertrophy but instead is linked to a higher degree of apoptosis and even less cardiac hypertrophy during chronic GPCR stimulation. These findings suggest that an upregulation of both TRPC4α and TRPC4β in the heart might activate hypertrophic signaling pathways but also pro-apoptotic mechanisms which promote cell fragility and cardiomyocyte death during chronic GPCR stimulation.

## Materials and Methods

### Cardiomyocyte isolation, cell culture and adenoviral infection

Neonatal rat cardiomyocytes (NRCs) were isolated from 1–3 day old Wistar rats (Charles River Laboratories). After the animals were sacrificed by decapitation, the hearts were quickly removed and rinsed with cold phosphate buffered saline (PBS). The subsequent steps of isolation were described previously [5] with some variations. Cardiomyocytes were cultured in minimal essential medium (MEM) including 5% fetal bovine serum (FBS) for 24 h, washed with PBS and cultured for additional 24 h in MEM/5% FBS when used for immunocytochemistry and biochemistry and in MEM/1% FBS for Ca^2+^ recordings to guarantee sparse cell density. The cells were infected with adenoviruses encoding beta galactosidase (βgal; Ad-βgal) [5], N-terminally myc-tagged human TRPC4α (Ad-C4α; NM_016179.2) [11], N-terminally myc-tagged murine TRPC4β (Ad-4β; NM_001253682.1) [11] and/or NFATC1-GFP (from Seven Hills Bioreagents (Cincinnati, USA; [12]). Adenoviruses were purified using the Adenovirus purification kit, Vivapure^®^ AdenoPACK^™^ (Sartorius) and the titer was determined by using the Quick Titer Adenovirus Titer ELISA Kit (CELL BIOLABS). The adenoviruses were applied in a MOI of 10 and applied in MEM/0% FBS for 4 hours. Expression is under the control of a CMV promoter. After that, the medium was changed to MEM/1%FBS for further 24 h. For the evaluation of agonist-induced hypertrophy and signaling, cells were serum starved for 24 h and stimulated with a combination of angiotensin II (Ang II; 100 nM)/phenylephrine (PE; 50 μM; Ang II/PE) or vehicle for 24 h.

### Transfection of siRNAs

To down-regulate endogenous TRPC4α we purchased pre-designed siRNAs from Life Technologies which target the bp 2577–2601 region of rat TRPC4α (TRPC4α sense: AACAGAGAAGAUUUGGUUUGCGUUU; TRPC4α antisense: AAACGCAAACCAAAUCUU CUCUGUU). As control we used Silencer^®^ Negative Control No. 1 siRNA. NRCs seeded on coverslips were transfected with the respective siRNAs using Lipofectamine RNAiMAX (Life technologies) according to the manufacturer’s instructions. After 48 h, the cells were stimulated with Ang II (100 nM) /PE (50 μM) for 24 h followed by immunocytochemistry and Western blotting.

### Transverse aortic constriction (TAC) surgery and echocardiography

8–12 week old mice (C57/BL/6J) were subjected to TAC or sham surgical procedure to induce chronic left ventricular pressure overload as described previously [13]. The animals were anesthetized with 1.5% isoflurane, intubated, and ventilated with a volume-cycled rodent respirator. The thorax was opened and TAC was achieved by ligating the aorta with a 7–0 Nylon thread on top of a 27-gauge needle. Apart from the aortic ligation, the sham animals underwent the same procedures. After closing the chest and skin, the animals were extubated and kept for 8 weeks. As pre-emptive pain medication, buprenorphine (0.05–0.1 mg/kg s.c.) was used and administered subcutaneously prior to thoracotomy.

Echocardiography was performed in a blinded manner using the Vevo 1100 high-resolution imaging system (VisualSonics) after 2 and 8 weeks of TAC/sham operation. During the procedure, mice were anesthetized with 2% isoflurane by inhalation. After the last echocardiographic measurements, the animals were sacrificed by cervical dislocation. Animal housing and chow were provided by the local animal facility of the University Hospital of Würzburg.

### Ethics statement

All animal experiments were approved by the authority of Unterfranken and the standing authority of the University Hospital of Würzburg (permit number: 55-2-2531.01-77/13).

No human subjects were used.

### Ca^2+^ measurements

NRCs were cultured on coverslips and loaded with Fura-2 (2 μM; Life technologies) in a Ca^2+^-free normal Tyrode solution (in mmol/L: NaCl 140, KCl 4, MgCl_2_ 1, Hepes 5, glucose 10; pH 7.4) for 15 min at room temperature (RT; [13]). Ca^2+^ transients were measured in normal Tyrode including 1 mM CaCl_2_ with or without 1 μM Ang II at a pacing frequency of 1 Hz.

To measure diastolic Ca^2+^ levels, cardiomyocytes were paced at 1 Hz in a 1 mM CaCl_2_ containing normal Tyrode. After a resting period of 20 s (R), the cells were perfused with a normal Tyrode containing 10 mM EGTA for 70 s (R_min_). After changing to normal Tyrode without NaCl (substituted with 140 mM LiCl) and 1 mM CaCl_2_, 10 μM ionomycin (Sigma Aldrich) was added (R_max_). After subtracting the background, the fluorescence ratio (R = F340/F380) was converted to [Ca^2+^]_i_ using the equation [Ca^2+^]_i_ = Kdβ(R−R_min_)/(R_max_−R); Kd = 236 nM; R_min_, R_max_, and β were determined experimentally [14,15]. To assess baseline TRPC4 channel activity, cells were perfused with a nominally Ca^2+^-free and then a 1.8 mM CaCl_2_ containing normal Tyrode to challenge ion channel activity. The buffers contained verapamil (10 μM; Sigma Aldrich) to block L-type Ca^2+^ channel (LTCC) activity.

The effect of Ang II on cytosolic Ca^2+^ levels in quiescent cardiomyocytes was determined by the acute application of Ang II. The cells were kept in Tyrode containing 1 mM CaCl_2_, 10 μM verapamil ± BTP2 (5 μM; Calbiochem) or DMSO, respectively. At the beginning of each experiment, cell viability was tested by eliciting Ca^2+^ transients at a pacing frequency of 1 Hz in a 1 mM CaCl_2_ containing Tyrode.

All Ca^2+^ measurements were performed using the “Myocyte and Contractility System” from Ionoptix. Data were corrected for background 340/380 fluorescence and analyzed using the IonWizard 6.3 software (Ionoptix).

### Immunocytochemistry, cell surface measurement and NFAT translocation

NRCs were cultured on coverslips coated with laminin (BD Sciences). After adenoviral infection or siRNA transfection, serum starvation and stimulation with Ang II/PE, cells were fixed in 4% paraformaldehyde at RT, washed with PBS, permeabilized with 0.5% Triton X-100, and blocked with a blocking solution (1% BSA, 0.1% cold water fish skin gelatin, 1% Tween-20 in PBS) for 1 h. Cells were then labeled using an antibody against α-actinin (Sigma-Aldrich) and a secondary antibody (AlexaFluor 568, Life Technologies) to visualize cardiomyocytes. 4′, 6-diamidine-2′-phenylindole dihydrochloride (DAPI) was used to visualize the nucleus (300 nM, 5 min, RT; Life Technologies). For detecting the translocation of NFAT, cardiomyocytes infected with NFATc1-GFP, were stimulated with AngII/PE (1 μM/50 μM) for 15 min and processed for further fluorescence recordings.

Fluorescence microscopy was performed using a Zeiss Axiovert 135 microscope with a 63x oil immersion objective (Zeiss Plan Neofluar). Quantification of NFATc1 nuclear translocation was determined by measuring the mean grey value of NFATc1-GFP in the nucleus and in the cytoplasm using Image J software. The quotient of nucleus to cytoplasm was used to compare the localization of NFATc1. Cell areas were determined using Image J software.

### Western blotting

Protein extracts from mouse hearts and NRCs were subjected to SDS-PAGE and Western blotting as described previously [5]. Immunoblots were blocked, treated with primary antibodies, secondary horseradish peroxidase-conjugated antibodies and developed using a chemiluminescence detection system [16]. As primary antibodies we used cleaved caspase-3 (Asp 175; Cell signaling, #9661;); glyceraldehyde 3-phosphate dehydrogenase (GAPDH, Millipore, #MAB374); histone deacetylase 4 (HDAC 4; Abcam, # ab1437); phospho-HDAC 4 (PS 632; Sigma Aldrich, #SAB4300155); myc-Tag (Cell signaling, #2272), phospholamban (PLN; Millipore, MA3-922); phospho-phospholamban (PT17, Badrilla Ltd, #A010-13AP); TRPC4 (Alomone labs, #A010-13AP).

### Caspase-3 activity

Caspase-3 activity was analyzed using the Caspase-Glo 3/7 Assay kit (Promega). NRCs were lysed in a buffer containing (mM) 150 NaCl, 50 Tris (pH 7.4), 0.5 DTT and 1% Triton-X-100, phosphatase (set II and IV; Calbiochem) and protease inhibitor cocktails (Roche). The protein concentration was adjusted to 0.2 mg/ml. 50 μl of the reaction reagent were added to 50 μl of sample in white 96 wells. After 1 h of incubation, luminescence was measured using a Perkin-Elmer 1420 Multilabel Counter.

### Cell viability assay

Cell viability was determined by using the trypan blue exclusion assay [17]. Trypan blue stains only dead or perforated cells. NRCs were isolated and seeded in a 6-well plate. After infection with different adenoviruses, serum starvation and stimulation with Ang II/PE, cells were harvested with trypsin and centrifuged. Supernatants were removed and pellets were re-suspended in MEM/0% FBS. 10 μl cell suspension were mixed with 10 μl of 0.40% trypan blue solution. The ratio of viable cells as a percentage of total cells was determined in a hemocytometer.

### Statistics

Data are presented as mean ± SEM. The two-tailed unpaired Student’s *t-*test was used to analyze significant differences between two groups. Significant differences between more than two groups were assessed by One-way ANOVA with Bonferroni *t*-test. Two-way ANOVA followed by Bonferroni *t*-test was used to detect significant differences when variables were dependent on two factors. All analyses were performed by using SigmaPlot 13. *P* ≤ 0.05 was considered significant.

## Results

### Up-regulation of TRPC4α and TRPC4β in the heart after TAC treatment

**Fig 1:**
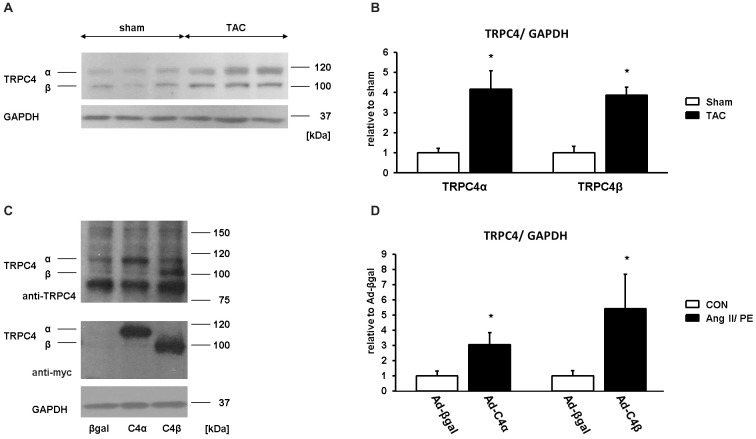
Enhanced expression of TRPC4α and TRPC4β after pressure overload-induced cardiac hypertrophy. A, Expression levels of TRPC4α and TRPC4β were analyzed in mouse heart homogenates from transverse aortic constriction (TAC) or sham treated mice (3 mice/group). B, quantification of TRPC4α and TRPC4β expression levels relative to sham controls. *P<0.05 vs Sham. C. Detection of TRPC4α and TRPC4β in lysates from neonatal rat cardiomyocytes with TRPC4 or myc-tag antibodies. D, Average quantified values of TRPC4α and TRPC4β signals expressed relative to Ad-βgal for n = 3 experiments. *P<0.05 vs Ad-βgal CON. Glyceraldehyde 3-phosphate dehydrogenase (GAPDH) was used as loading control.

For most of the TRPC subunits, low expression levels have been found in the heart which increase after pathological stimulation by TAC or MI treatment [2,18–20]. In order to find out whether individual TRPC4 splice variants show changes in their expression profile we subjected mice to 8 weeks of TAC or sham surgery. As shown in Fig 1A, both TRPC4 isoforms can be detected in the heart, with TRPC4α at ~112 kDa and TRPC4β at ~102 kDa. TAC treatment induced an increased expression of both isoforms compared to sham controls (Fig 1B).

To further characterize functional effects of elevated TRPC4α or TRPC4β expression levels, we infected rat cardiomyocyte cultures with adenoviruses encoding human TRPC4α (Ad-C4α) or mouse TRPC4β (Ad-C4β), respectively. Expression of TRPC4α and TRPC4β was detected with both anti-myc and anti-TRPC4 antibodies (Fig 1C) and was significantly elevated compared to control-infected (Ad-βgal) cardiomyocytes (Fig 1D).

### Both TRPC4α and TRPC4β show spontaneous activity in cardiomyocytes

**Fig 2:**
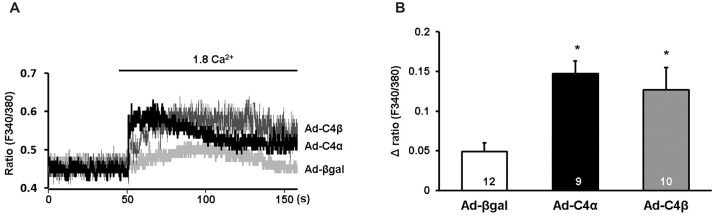
Spontaneous Ca^2+^ influx in TRPC4α and TRPC4β overexpressing neonatal rat cardiomyocytes. A, Representative Fura-2 Ca^2+^ recordings of neonatal rat cardiomyocytes (NRCs) infected with either Ad-βgal, Ad-TRPC4α or Ad-TRPC4β. Cells were perfused with a nominally Ca^2+^-free Tyrode solution including verapamil (10 μM). After 50 s, Ca^2+^ influx was challenged by perfusing the cells with a solution containing 1.8 mM Ca^2+^. B, Mean Δ ratio values were calculated by subtracting the peak value at 1.8 mM from the baseline value at 0 mM Ca^2+^.*P<0.05 vs Ad-βgal. Cells from three different experiments were analyzed. N-number of cells is indicated in the bars.

Since spontaneous channel activity is a common feature among TRPC channels [21–23] we first analyzed whether the overexpression of TRPC4α and TRPC4β would alter cytosolic Ca^2+^ signals in NRCs. To assess baseline TRPC4 activity, we perfused NRCs with a nominally Ca^2+^-free Tyrode buffer after which we added 1.8 mM Ca^2+^ (Fig 2A). The rise of cytosolic Ca^2+^ after Ca^2+^ re-addition was significantly elevated in cells overexpressing TRPC4α and TRPC4β but was barely detectable under control conditions (Fig 2B). After reaching a plateau phase, the cytosolic Ca^2+^ levels decreased after 50–150 s of Ca^2+^ re-addition. LTCC-dependent effects on the Ca^2+^ signals were inhibited by the addition of verapamil (10 μM) during the measurements.

### TRPC4β is more sensitive to Ang II stimulation than TRPC4α in neonatal rat cardiomyocytes

**Fig 3:**
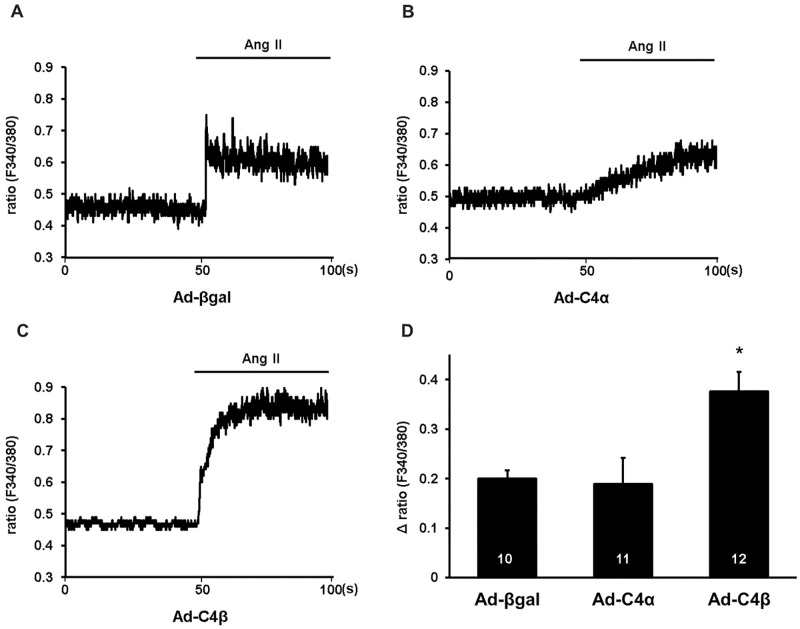
TRPC4β overexpression is connected with an increased Ca^2+^ influx after angiotensin II stimulation of cardiomyocytes. A, Representative Fura-2 Ca^2+^ traces in neonatal rat cardiomyocytes (NRCs) infected with either Ad-βgal (A), Ad-TRPC4α (B) or Ad-TRPC4β (C). Quiescent cells were perfused with a normal Tyrode solution containing 1 mM Ca^2+^. Verapamil (10 μM) was included throughout the recordings. After 50 s, angiotensin II (Ang II; 1 μM) was applied. D, Mean Δ ratio values were calculated by subtracting the peak value after Ang II addition from the baseline value. *P<0.05 vs Ad-βgal and Ad-TRPC4α. Cells from three different experiments were analyzed. N-number of cells is indicated in the bars.

TRPC channels are well-known downstream targets of the GPCR-PLC signaling pathway in various cell types including cardiomyocytes [1]. As a next step, we therefore analyzed the effect of GPCR stimulation on TRPC4α and TRPC4β-dependent Ca^2+^ signals in NRCs. Control and Ad-C4α and Ad-C4β-infected cells were perfused with 1 mM Tyrode for 50 s and treated with Ang II (1 μm) in the presence of verapamil (10 μM). In control infected cells, Ang II stimulation caused a slight Ca^2+^ increase (Fig 3A). The amplitude of the Ca^2+^ influx in TRPC4α-infected cardiomyocytes was comparable to controls. However, the Ca^2+^ entry seemed to increase more slowly despite the similar Ca^2+^ plateau (Fig 3B). In comparison to control and Ad-C4α-infected NRCs, the Ca^2+^ elevation in TRPC4β overexpressing cells was markedly enhanced (Fig 3C and 3D) which might indicate a higher sensitivity of TRPC4β to Ang II stimulation.

As another approach to show that TRPC4 channels are implicated in the Ang II-dependent Ca^2+^ influx, we pre-treated NCRs with the non-selective TRPC and Ca^2+^ release-activated channel (CRAC) blocker, the 3,5-bis (trifluoromethyl) pyrazole derivate, BTP2 (5 μM; S1 Fig) [24,25]. Pre-treatment with BTP2 resulted in a significantly attenuated Ca^2+^ influx in control-infected NRCs. The inhibitory effect of BTP2 was similar between control, Ad-C4α and Ad-C4β-infected cells.

### Ca^2+^ transient characteristics of cardiomyocytes overexpressing TRPC4α and TRPC4β

**Fig 4:**
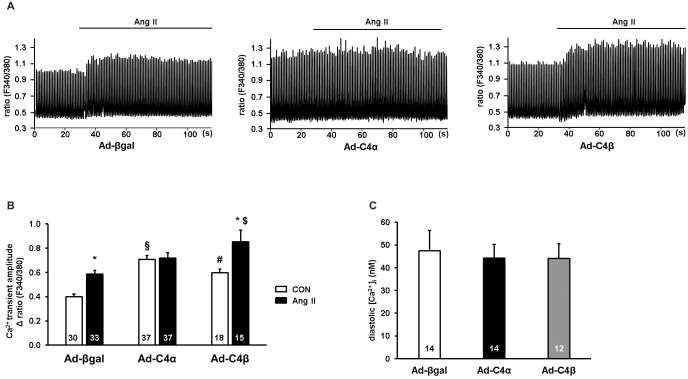
Overexpression of TRPC4α increases the Ca^2+^ transient amplitude at baseline and TRPC4β elevates the Ca^2+^ transient amplitude at baseline and during Ang II stimulation. A, Ca^2+^ transients were measured in Ad-βgal, Ad-C4α or Ad-C4β neonatal rat cardiomyocytes (NRCs) in a normal Tyrode solution with angiotensin II (Ang II; 1 μM) or without Ang II during 1 Hz of pacing. B, The Ca^2+^ amplitude under steady state pacing was significantly increased in Ad-C4α and Ad-C4β cardiomyocytes. Ang II stimulation resulted in an elevation of the Ca^2+^ peak in Ad-βgal and Ad-C4β but not Ad-C4α cardiomyocytes as analyzed after 30–60 s of Ang II application. *P<0.05 vs Ad-βgal CON and Ad-C4β CON; ^#^*P*<0.05 vs Ad-βgal CON; ^§^*P*<0.05 vs Ad-βgal and Ad-C4β CON; ^$^*P*<0.05 vs Ad-βgal Ang II/PE. C, Diastolic [Ca^2+^]_i_ levels measured with Fura-2 were not different between Ad-βgal, Ad-C4α or Ad-C4β NRCs. Cells from at least three different experiments were analyzed. N-number of cells is indicated in the bars.

TRPC channels have been found to affect Ca^2+^ cycling during excitation contraction-coupling (ECC) as well as contractility in cardiomyocytes [2,7]. In order to test whether contractile Ca^2+^ signals could be affected by the activity of TRPC4 splice variants, we measured Ca^2+^ transients in beating cardiomyocytes infected with Ad-C4α or Ad-C4β. During steady state pacing at 1 Hz (CON), it was noticeable that cardiomyocytes overexpressing TRPC4α and TRPC4β responded with an enhanced Ca^2+^ amplitude (Fig 4A and 4B) which was more pronounced in the presence of TRPC4α. This effect could be due to the spontaneous activity of TRPC4α and TRPC4β (Fig 2). In order to examine the responsiveness of the Ca^2+^ transients to GPCR stimulation during electric pacing, we again stimulated cardiomyocytes with Ang II (1 μM; Fig 4A and 4B). The administration of Ang II resulted in an enhanced Ca^2+^ amplitude in control-infected (Ad-βgal) NRCs after 30–60 s of Ang II stimulation. Interestingly, Ang II did not affect the transient amplitude when TRPC4α was overexpressed but caused a significant increase in the presence of TRPC4β. We also measured diastolic [Ca^2+^]_i_ with Fura-2, however we did not find detectable differences between control-infected, TRPC4α and TRPC4β overexpressing NRCs.

These findings suggest that TRPC4α increases Ca^2+^ transients at baseline, but does not affect Ca^2+^ transients during GPCR stimulation. The overexpression of TRPC4β on the other hand affects the Ca^2+^ transient amplitude at baseline and is connected with elevated Ca^2+^ transients during GPCR stimulation.

### Assessment of calcineurin/NFAT signaling in cardiomyocytes

**Fig 5:**
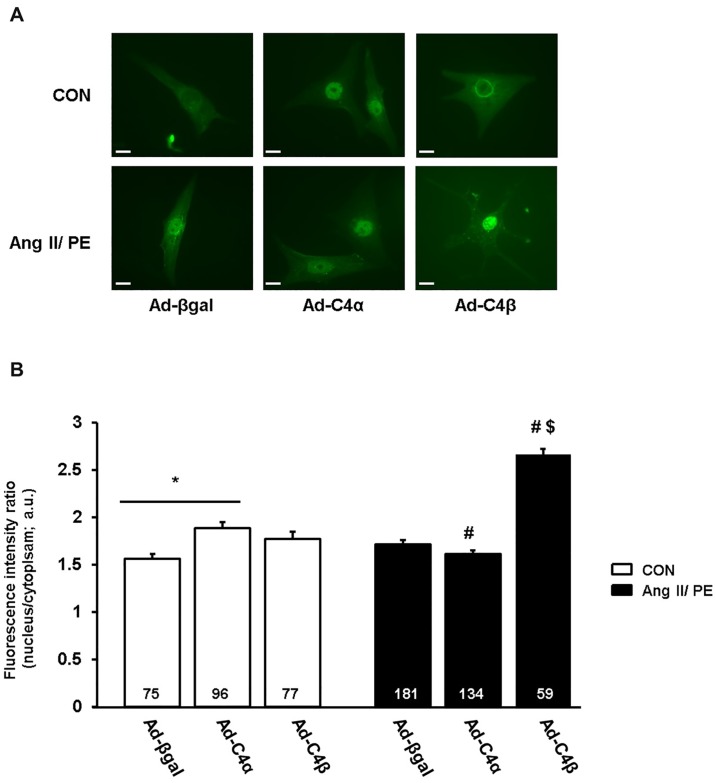
NFATc1 nuclear translocation is promoted by TRPC4α at baseline and by TRPC4β upon agonist stimulation. A, Neonatal rat cardiomyocytes (NRCs) were co-infected with Ad-βgal, Ad-TRPC4α or Ad-TRPC4β/ NFATc1-GFP. Nuclear localization of NFATc1-GFP was used as an indicator for its activation. Green: NFATc1-GFP. Scale bar: 20 μm. B, Quantification of NFATc1 localized in the nucleus of NRCs that were stimulated with angiotensin II/phenylephrine (Ang II/PE; 1 μM/50 μM) or vehicle (CON). *P<0.05 vs unstimulated; ^#^*P*<0.05 vs Ad-TRPC4α or Ad-TRPC4β CON, respectively; ^$^*P*<0.05 vs Ad-βgal and Ad-TRPC4α Ang II/PE. Cells from at least three different experiments were analyzed. N-number of cells is indicated in the bars.

A series of studies has shown TRPC channel-mediated Ca^2+^ signals that activate the pro-hypertrophic calcineurin/NFAT signaling pathway [1]. Accordingly, we were interested to find out whether TRPC4α and TRPC4β-mediated effects on cytosolic Ca^2+^ signals were translated in an altered calcineurin activity. As surrogate of calcineurin activation, we assessed the translocation of the transcription factor NFATc1 from the cytosol into the nucleus [26]. As shown in Fig 5A, NFATc1 is mainly detectable in the cytoplasm of Ad-βgal-infected NRCs. Interestingly, Ad-C4α-infected cardiomyocytes showed an increase of the nuclear NFAT localization at baseline (Fig 5A and 5B). We stimulated the cells with a combination of the GPCR agonists Ang II/PE for 15 min which resulted in an increasing but not significant nuclear localization of NFATc1 in Ad-βgal NRCs (Fig 5B). This effect on calcineurin/NFAT signaling in response to GPCR stimulation was clearly enhanced in cardiomyocytes overexpressing TRPC4β. In TRPC4α overexpressing NRCs, the activity of calcineurin/NFAT was not further augmented after Ang II/PE stimulation (Fig 5A and 5B).

### TRPC4α and TRPC4β do not enhance cardiomyocyte hypertrophy during agonist stimulation

**Fig 6:**
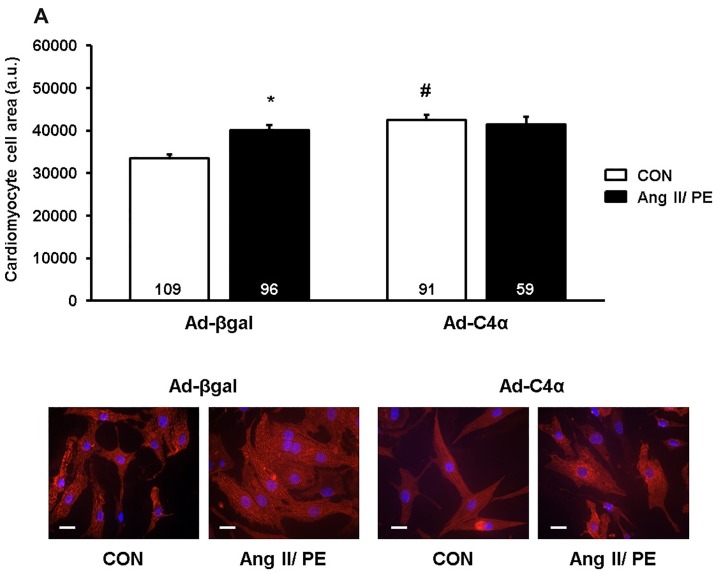
TRPC4α increases cardiomyocyte hypertrophy at baseline but not after GPCR stimulation. A, B Hypertrophic growth of cardiomyocytes was measured as an increase of the cell surface after angiotensin II/phenylephrine (Ang II/PE) or vehicle (CON) treatment for 24 h. Red: α-actinin; Blue: DAPI. *P<0.05 vs Ad-βgal CON; ^#^*P*<0.05 vs Ad-βgal CON. Cells from at least three different experiments were analyzed. Scale bar: 20 μm. N-number of cells is indicated in the bars.

To further explore whether TRPC4α and TRPC4β-mediated effects on Ca^2+^ cycling and signaling were reflected by changes in the hypertrophic response, we monitored the hypertrophic growth of cardiomyocytes in an agonist-mediated approach. Cardiomyocytes were infected with Ad-C4α, Ad-C4β or Ad-βgal, serum starved and stimulated with Ang II/PE or vehicle in serum free conditions for 24 h. Ang II/PE treatment resulted in a typical enlargement of the cell surface in Ad-βgal NRCs (Fig 6A). Interestingly, TRPC4α induced a hypertrophic growth at baseline but did not further promote the hypertrophic growth after agonist stimulation (Fig 6A and 6B).

**Fig 7:**
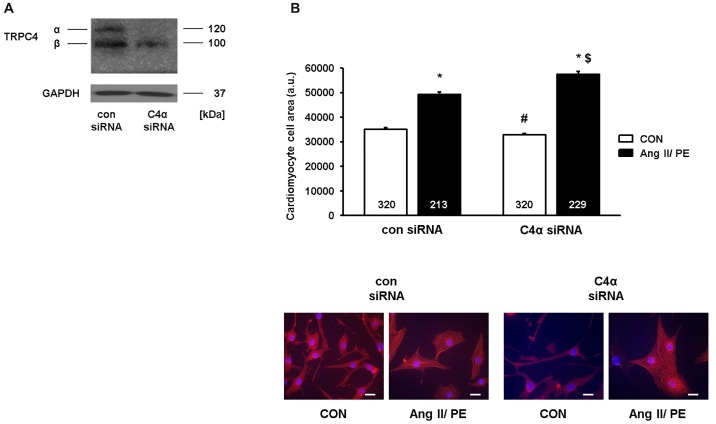
Down-regulation of endogenous TRPC4α decreases baseline cardiomyocyte size and promotes cardiomyocyte hypertrophy after chronic agonist stimulation. A, TRPC4α was down-regulated in neonatal rat cardiomyocytes (NRCs) by a siRNA-dependent approach. Down-regulation of TRPC4α was verified by Western blotting. B, Hypertrophic growth of NRCs transfected with control siRNA (con siRNA) or TRPC4α (C4α) siRNA was measured as an increase of the cell surface after angiotensin II/phenylephrine (Ang II/PE) or vehicle (CON) treatment for 24 h. Red: α-actinin; Blue: DAPI. *P<0.05 vs con siRNA and C4α siRNA CON; ^#^*P*<0.05 vs con siRNA CON; ^$^*P*<0.05 vs con siRNA Ang II/PE. Cells from three different experiments were analyzed. Scale bar: 20 μm. N-number of cells is indicated in the bars.

We further pursued the cell size analyses by applying a siRNA-mediated knock-down strategy. TRPC4α and TRPC4β differ within a C-terminal 252 AA stretch which allowed us to specifically knock-down TRPC4α in NRCs (Fig 7A). Intriguingly, this maneuver caused the opposite effect of Ad-C4α and resulted in less baseline cardiomyocyte hypertrophy but an increased hypertrophic enlargement after chronic Ang II/PE stimulation compared to Ad-βgal-infected cells (Fig 7B).

**Fig 8:**
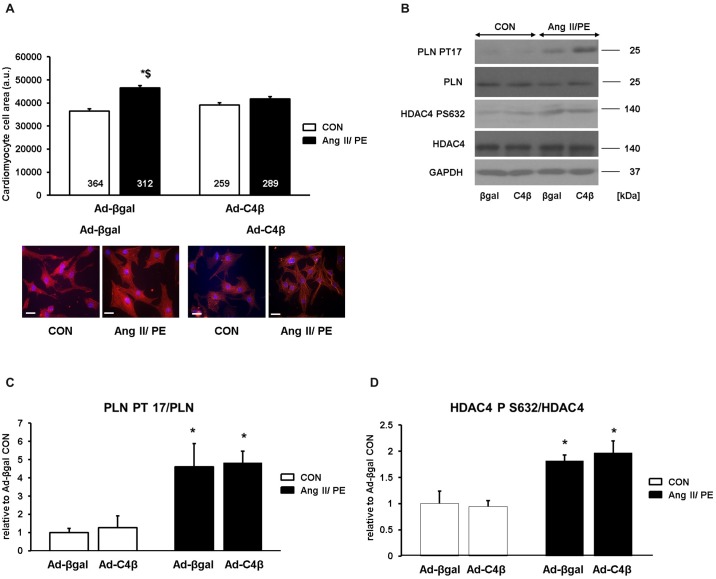
TRPC4β attenuates hypertrophic growth in cardiomyocytes after GPCR stimulation. A, Hypertrophic growth of cardiomyocytes was measured as an increase of the cell surface after angiotensin II/phenylephrine (Ang II/PE) or vehicle (CON) treatment for 24 h. Red: α-actinin; Blue: DAPI. *P<0.05 vs Ad-βgal CON; ^$^*P*<0.05 vs Ad-C4β AngII/PE. Cells from three different experiments were analyzed. Scale bar: 20 μm. N-number of cells is indicated in the bars. B, Expression and phosphorylation of phospholamban (PLN) and histone deacetylase 4 (HDAC4) in Ad-βgal and Ad-TRPC4β NRCs after stimulation with Ang II/PE or vehicle (CON) for 24 h. phospho-PLN: PLN PT17; phospho-HDAC4: HDAC4 PS632; glyceraldehyde 3-phosphate dehydrogenase: GAPDH. Shown are representative immunoblots from three different experiments. C, Average quantified values of PLN PT17 signals expressed relative to Ad-βgal CON for n = 3 experiments. D, Average quantified values of HDAC4 PS632 signals expressed relative to Ad-βgal CON for n = 3 experiments.

To complement our findings we also examined the regulatory role of TRPC4β in the hypertrophic response. TRPC4β did not cause baseline hypertrophy as found for TRPC4α but similar to TRPC4α failed to cause an increase in hypertrophy after agonist stimulation (Fig 8A) despite its effect on NFAT activation after GPCR stimulation. We therefore examined the activity of the Ca^2+^-calmodulin-dependent protein kinase II (CamKII) as alternative Ca^2+^-dependent mechanism which might counteract a calcineurin/NFAT-mediated hypertrophic effect. To indirectly assess the activity of CamKII we examined the phosphorylation status of PLN (Thr 17) and HDAC4 (Ser 632) in cardiomyocytes lysates from TRPC4-infected cardiomyocytes (Fig 8B). Both PLN (Fig 8C) and HDAC (Fig 8D) phosphorylation were greater after chronic GPCR stimulation but did not differ between Ad-C4β and Ad-βgal NRCs.

### Overexpression of TRPC4α and TRPC4β enhances apoptosis and impairs cardiomyocyte survival during chronic agonist treatment

**Fig 9:**
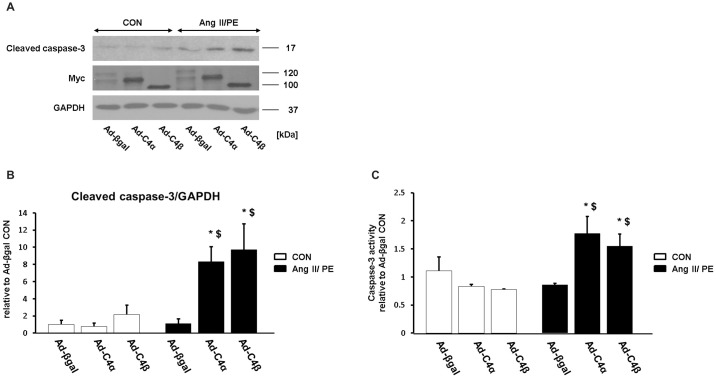
Increased apoptotic rate in cardiomyocytes overexpressing TRPC4α or TRPC4β. A, Cleaved caspase-3 expression levels were detected in neonatal rat cardiomyocytes (NRCs) after Ang II/PE or vehicle (CON) treatment. GAPDH: loading control. Myc: detection of TRPC4α and TRPC4β. Shown are representative immunoblots from three independent experiments. B, Average quantified values of cleaved caspase-3 expression levels relative to Ad-βgal CON for n = 3 experiments. C, Capase-3 activity was compared between Ad-βgal, Ad-TRPC4α and Ad-TRPC4β in NRCs. Caspase-3 assays were performed in duplicate and average values were quantified relative to Ad-βgal CON; n = 3 experiments.

We further explored whether the overexpression of TRPC4α and TRPC4β enhances apoptosis rather than hypertrophy during chronic Ang II/PE stimulation. We therefore detected caspase-3 levels by Western blotting (Fig 9A and 9B) and measured the activity of caspase-3 (Fig 9C) using cardiomyocyte homogenates. TRPC4α- and TRPC4β-infected cardiomyocytes showed a higher caspase-3 activity compared to control-infected cardiomyocytes.

**Fig 10:**
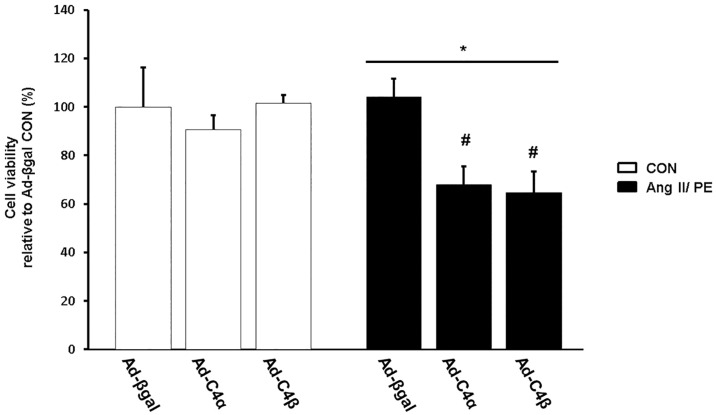
TRPC4α and TRPC4β lower cardiomyocyte viability after chronic agonist stimulation. The vitality of cardiomyocytes expressing βgal (Ad- βgal), TRPC4α (Ad-C4α) or TRPC4β (Ad-C4β) was determined after angiotensin II/phenylephrine (Ang II/PE) or vehicle (CON) treatment for 24 h by applying the trypan blue exclusion assay. The assay was performed in duplicate and average values were quantified relative to Ad-βgal CON; n = 3 experiments. *P<0.05 vs Ad-C4α and Ad-C4β Ang II/PE. B. ^#^*P*<0.05 vs Ad-C4α and Ad-C4β CON.

Up to this point our data indicate that TRPC4α and TRPC4β fail to induce a hypertrophic growth of cardiomyocytes during chronic Ang II/PE stimulation but show a higher sensitivity to apoptosis. Given this unexpected finding, we further analyzed whether enhanced TRPC4 expression levels could result in an impaired cardiomyocyte viability rather than increased hypertrophy during agonist stimulation. To follow this presumption that TRPC4 could be critical for cardiomyocyte vitality we examined the cell viability by a trypan blue exclusion assay. Under vehicle-treated conditions (CON; Fig 10A), there was no difference between Ad-C4α, Ad-C4β or Ad-βgal-infected cardiomyocytes. Ang II/ PE stimulation did not affect the viability of control (Ad-βgal) cardiomyocytes but caused a marked loss of viability of TRPC4α or TRPC4β-infected cardiomyocytes.

## Discussion

The potency of TRPC channels on Ca^2+^ handling and downstream signaling events in cardiomyocytes is probably based on a complex interplay between a variety of factors including the expression level, activity and cellular localization of certain TRPC subunits, the heteromerization status, the type of disease and its respective stimuli. In the present study we hypothesized that TRPC channel activity and their effects on cardiomyocyte function are also dependent on select TRPC splice variants.

Our first aim was to examine whether an upregulation of TRPC4α and TRPC4β as observed after TAC treatment would alter cardiomyocyte Ca^2+^ signals, signaling and hypertrophy. When analyzing Ca^2+^ transients, it became evident that overexpression of the human TRPC4α isoform resulted in elevated Ca^2+^ transient amplitudes which might result from the basal activity of TRPC4α. However, while the Ca^2+^ transients in control and TRPC4β-infected cardiomyocytes increased after Ang II application, there was no further Ca^2+^ elevation noticeable in the presence of TRPC4α. It seemed that human TRPC4α exhibited some degree of insensitivity to GPCR stimulation in neonatal rat cardiomyocytes, a characteristic which has already been found in HEK cells [8,23]. Also in quiescent myocytes, the Ang II-elicited Ca^2+^ influx was not different to controls and even reached the plateau phase with slower kinetics compared to control and TRPC4β-infected cells. In this respect, PDZ-containing scaffolding proteins have been found to affect activation kinetics of TRPC channels. For example, histamine-elicited TRPC5 currents were prolonged by the PDZ-containing scaffolding protein ezrin-binding phosphoprotein-50 (EBP50 [27]). EBP50 might exert its modulatory function by binding the C terminal VTTRL domain which is present in both TRPC5 and TRPC4 channels [28]. In neonatal rat cardiomyocytes, the PDZ-domain containing scaffolding protein shank3 was suggested to exclusively tether TRPC4α to PLCβ1b in the plasma membrane. Although we do not have proof, it is likely that this association affects functional properties of TRPC4α becoming manifest as lower sensitivity and activation kinetics after GPCR stimulation. Although TRPC4β exhibits a putative PDZ binding motif in its C terminus, it was not found to be complexed by shank3. This lack of interaction might be due to the missing 84 AA stretch in the C terminus of TRPC4β which has been described as important protein/lipid binding and regulatory domain. [8,10].

Regulatory properties of different TRPC4 isoforms might also be dependent on the species. For example, human TRPC4α has been found to be barely stimulated by GPCR agonists [8,23], which otherwise increase the activity of rodent TRPC4α [8]. Thus, species-dependent variations might also affect the results of the present study and could explain the insensitivity of human TRPC4α to GPCR stimulation: the rise of the Ca^2+^ amplitude and its prevention during Ang II stimulation, the unchanged translocation of NFATc1 after acute GPCR stimulation. Finally, cardiomyocytes overexpressing TRPC4α showed an elevated hypertrophic enlargement but were not able to hypertrophy after chronic agonist stimulation. As mentioned above, this type of baseline hypertrophy has been suggested by a recent study proposing a complex formation between TRPC4α and PLCβ1 as part of the underlying pro-hypertrophic potential of TRPC4α [11]. Our results do not rule out such a possibility but further show that TRPC4α exhibits constitutive activity and enhances calcineurin/NFAT signaling which might substantially influence this initial hypertrophic effect. Importantly, downregulation of TRPC4α had the opposite effect to TRPC4α overexpression. The cardiomyocyte size was smaller at baseline but enlarged after 24 h of agonist stimulation. These findings support the notion that the spontaneous activity of TRPC4α creates a pre-existing hypertrophic phenotype which is not further augmented, but rather is coupled to an accelerated apoptotic cardiomyocyte death during chronic GPRC stimulation. The transition from hypertrophy to apoptosis might be based on pro-apoptotic signaling proteins downstream of the GPCR signaling pathway. For example, p38 and c-Jun NH_2_-terminal kinases (JNK) have been found as crucial pro-death signaling proteins responding to high level Gαq activation [29] and could balance hypertrophy and apoptosis in a cross-talk with the calcineurin/NFAT signaling pathway in the present study. In this context it is interesting to note that pre-activated calcineurin might activate the apoptosis signal-regulating kinase 1 (ASK1) and its downstream targets p38 and JNK which then phosphorylate and inactivate NFAT [30]. In TRPC4α overexpressing cardiomyocytes this negative feedback regulation might become manifest during chronic GPCR stimulation and limit hypertrophic gene expression, prevent hypertrophic development but increase the susceptibility for cardiomyocyte apoptosis. However, it has to be kept in mind that although the nuclear localization of NFATc1 was significantly less in stimulated versus non-stimulated Ad-C4α cardiomyocytes, it was still similar between Ad-βgal and Ad-C4α infected cardiomyocytes (no signs of cell death in control treated cardiomyocytes under stimulatory conditions). This might suggest that although p38/JNK-dependent counter-regulation might occur, other or additional mechanisms are involved in the prevention of cell growth and induction of apoptosis in Ad-C4α cardiomyocytes during GPCR stimulation. For example, it might be assumed that calcineurin plays a dual role by linking a TRPC4α-mediated Ca^2+^ entry to hypertrophy at baseline but to apoptotic signaling pathways under stimulatory conditions. In this context it is interesting to note that both apoptotic and hypertrophic functions have been described for calcineurin in cardiomyocytes [31,32].

An increased apoptotic rate was also noticeable in cardiomyocytes overexpressing TRPC4β, although the preceding processes seemed to be different to Ad-C4α NRCs. Similar to TRPC4α, TRPC4β allowed an enhanced Ca^2+^ influx and increased the Ca^2+^ transient amplitude at baseline. Paradoxically, the diastolic Ca^2+^ levels in both TRPC4α and TRPC4β over-expressing NRCs were not altered. It is possible that a TRPC4-mediated Ca^2+^ overload might be compensated by an enhanced Ca^2+^ uptake in the SR which would also explain the increased Ca^2+^ transient amplitudes in Ad-C4α and Ad-C4β NRCs.

In contrast to TRPC4α, TRPC4β over-expression was also connected with a marked elevation of the Ca^2+^ transient amplitude during Ang II stimulation. This might indicate that Ang II stimulation evokes a TRPC4β-mediated Ca^2+^ influx that contributes to elevated systolic Ca^2+^ peaks. The sensitivity of TRPC4β towards GPCR stimulation was also noticeable as a pronounced Ca^2+^ influx in quiescent Ad-C4β-infected cardiomyocytes. A GPCR-dependent regulation is a well-described characteristic of TRPC4β across different species [8], and according to our data this is also true in the setting of neonatal rat cardiomyocytes.

We also analyzed the Ang II-dependent Ca^2+^ influx in the presence of the non-selective TRPC inhibitor BTP2. In NRCs of all three groups (Ad-βgal, Ad-C4α, Ad-C4β), the Ca^2+^ influx was blocked to a similar extent. However, it has to be kept in mind that BTP2 also blocks CRAC channels as suggested for other cell types [25,33]. Due to the lack of information regarding the precise inhibitory effect of BTP2, our data only propose TRPC4 channels as down-stream targets of Ang II stimulation, but do not reveal the precise mode of channel activation. A recent study suggests that TRPC1, 4 and 5 comprise SOC channels in association with ORAI1 that are downstream targets of aldosterone stimulation in NRCs [34]. Further studies are necessary to examine whether such a miscellaneous constellation could also mediate Ca^2+^ responses after Ang II stimulation.

Given the Ca^2+^ characteristics of TRPC4β overexpressing NRCs, we expected a pronounced hypertrophic cardiomyocyte growth under baseline as well as stimulatory conditions. However, it came as a surprise that the TRPC4β-mediated baseline Ca^2+^ influx and the elevated Ca^2+^ transients were not coupled to an increased calcineurin/NFAT activation and hypertrophic growth as found for TRPC4α. This discrepancy might be influenced by intrinsic structural and functional differences between TRPC4α and TRPC4β, as well as by distinct interactions with protein partners such as PLCβ1b [11] that regulate the properties of TRPC4 channels and their effect on baseline cardiomyocyte physiology.

Even more puzzling was the fact that under stimulatory conditions, an elevated TRPC4β-mediated Ca^2+^ influx was coupled to an enhanced calcineurin/NFAT activity, however an attenuated hypertrophic cardiomyocyte growth. We instantly assumed that the inefficiency of TRPC4β to alter cardiomyocyte remodeling during GPCR stimulation might be simply based on a reduced activity of other Ca^2+^-dependent signaling proteins, such as CamKII. We found no obvious changes in the phosphorylation status of the CamKII-sensitive downstream targets, PLN or HDAC4, however, an enhanced caspase activity accompanied with a lower cardiomyocyte viability after chronic agonist treatment. Thus, during GPCR stimulation TRPC4β might activate calcineurin which is not necessarily connected with pro-hypertrophic but rather pro-apoptotic effects. Our findings also suggest that both TRPC4α and TRPC4β initiate apoptotic processes during chronic agonist stimulation in pathological conditions irrespective of their sensitivity to GPCR signaling.

Pro-apoptotic effects in cardiomyocytes have been described for other TRPC family members, such as TRPC7 which mediates apoptosis through the activation of calcineurin during Ang II stimulation [35]. However, in our study the prominent increase of nuclear NFATc1 localization might rule out pure apoptotic mechanisms in cardiomyocytes overexpressing TRPC4β. It might rather be speculated that during GPCR stimulation competing forces between hypertrophic and apoptotic mechanisms might be initiated during GPCR stimulation with a final domination of apoptosis and cell fragility. A similar phenomenon might occur in Ad-C4α NRCS during GPCR stimulation, with the one slight difference that TRPC4α induces the activation of calcineurin and NFAT and causes a pre-existing hypertrophy at baseline. This alteration might trigger apoptotic processes during GPCR stimulation which then limit but not reduce (as in cardiomyocytes overexpressing TRPC4β) a further progression of hypertrophy.

In summary our findings indicate that, although TRPC4α and TRPC4β might assemble to ion channels with different regulatory characteristics, their activity converges into a common cardiomyocyte phenotype which is more vulnerable to apoptosis during GPCR stimulation. In a bigger context these results might show that an upregulation of TRPC4α and TRPC4β could be a critical alteration contributing to cardiac disease progression.

## Supporting Information

S1 Fig Pre-treatment with the non-selective cation entry blocker BTP2 results in a reduced Ca^2+^ influx after angiotensin II stimulation. Representative Fura-2 Ca^2+^ traces in neonatal rat cardiomyocytes (NRCs) infected with either Ad-βgal (A), Ad-TRPC4α (B) or Ad-TRPC4β (C). Quiescent cells were perfused with a normal Tyrode solution containing 1 mM Ca^2+^ + BTP2 (5 μM). Verapamil (10 μM) was included throughout the recordings. After 50 s, angiotensin II (Ang II; 1μM) was applied.
(TIF)
