# Supplementary figures and images for: TRPC4α and TRPC4β Similarly Affect Neonatal Cardiomyocyte Survival during Chronic GPCR Stimulation
Source: PLoS One. 2016 Dec 19;11(12):e0168446. doi: 10.1371/journal.pone.0168446 (PMC5167390; doi:10.1371/journal.pone.0168446)

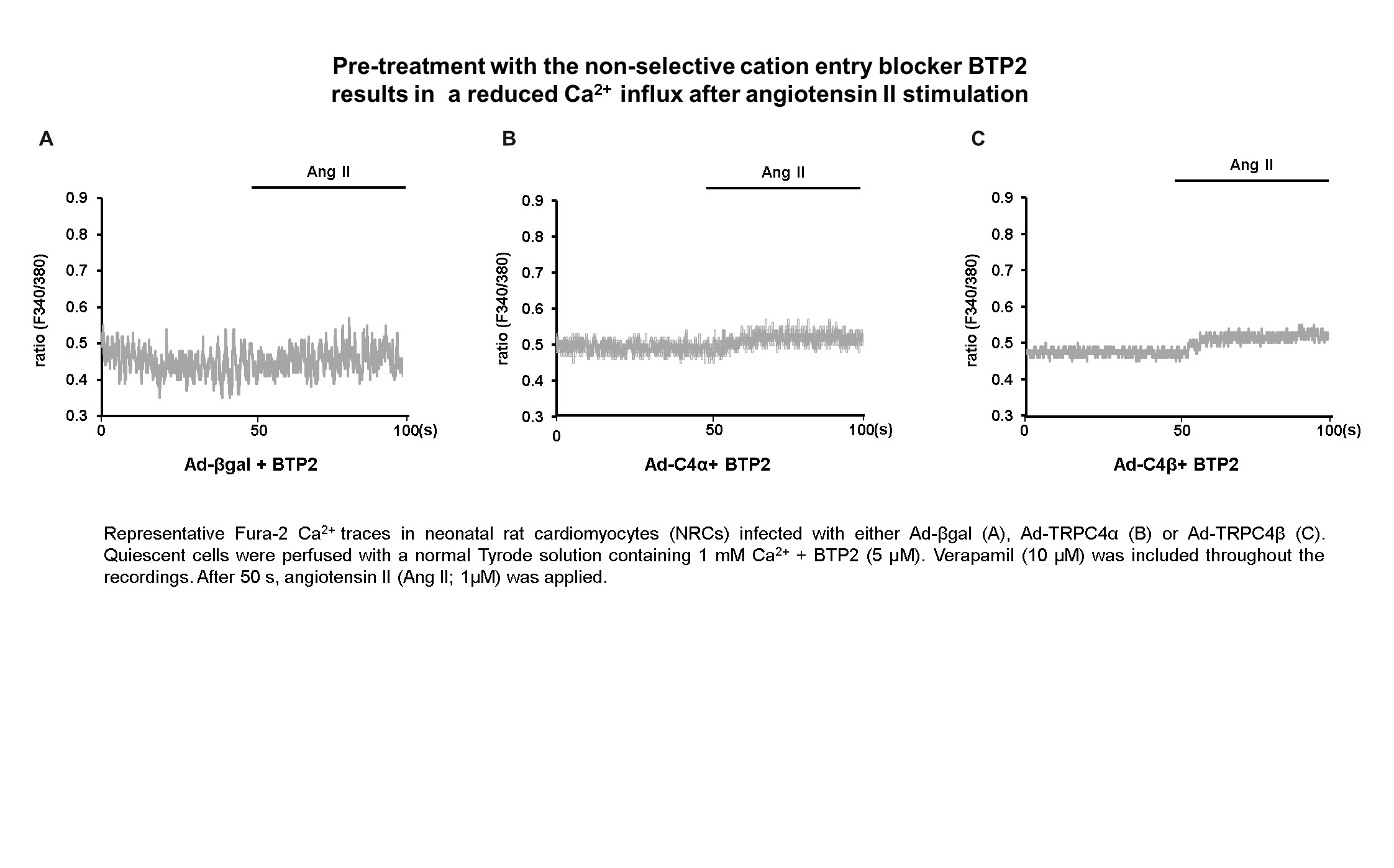

Supplement: S1 Fig — Representative Fura-2 Ca2+ traces in neonatal rat cardiomyocytes (NRCs) infected with either Ad-βgal (A), Ad-TRPC4α (B) or Ad-TRPC4β (C). Quiescent cells were perfused with a normal Tyrode solution containing 1 mM Ca2+ + BTP2 (5 μM). Verapamil (10 μM) was included throughout the recordings. After 50 s, angiotensin II (Ang II; 1μM) was applied. (TIF) [file pone.0168446.s001.tif]
